# Real-World Treatment Patterns in Men with Castration- Resistant Prostate Cancer Receiving Docetaxel

**DOI:** 10.36469/9894

**Published:** 2015-01-06

**Authors:** Keith L. Davis, Benjamin Gutierrez, Teresa Zyczynski, James A. Kaye

**Affiliations:** 1 RTI Health Solutions, Research Triangle Park, NC, USA; 2 Otsuka America Pharmaceutical Inc., Princeton, NJ, USA; 3 Bristol-Myers Squibb, Princeton, NJ, USA; 4 RTI Health Solutions, Waltham, MA, USA

**Keywords:** medical record review, treatment patterns, docetaxel, castration-resistant prostate cancer

## Abstract

**Background:** Docetaxel has been a standard of care for castration-resistant prostate cancer (CRPC) in the United States since 2004, yet little has been reported on its patterns of use in routine practice. To help understand these patterns, a retrospective study was conducted and is reported here.

**Methods:** Medical records from 394 patients treated in the United States were reviewed. Data were collected by 48 physicians from oncology (patient N=344) and 8 physicians from urology (patient N=50) practices. Inclusion criteria were: CRPC diagnosed between 2004 and 2010; received docetaxel; discontinued docetaxel due to rising prostate-specific antigen (PSA), progression of bone lesions, or progression of nodal or visceral metastases. Data were collected from physicians using an internet-based case report form. We evaluated patient demographics, characteristics of the docetaxel regimen, and other treatments used until docetaxel discontinuation.

**Results:** Patients had a mean [±SD] age of 66.5 [8.9] years, the majority (63%) were white, and geographic dispersion was similar to the US population. The majority of patients initiated docetaxel between 2008 and 2010. After CRPC diagnosis, 8% of patients had initiated another cancer-directed therapy before starting docetaxel. Most (78.9%) patients initiated docetaxel with prednisone, while 18.5% initiated docetaxel alone and 2.6% initiated with other medications. Half of patients initiated docetaxel within 1 month after CRPC diagnosis, while 25% started ≥6 months later. Other non-chemotherapy treatments used with docetaxel were hormonal therapy (22.8%), radiotherapy (17.3%), and surgery (4.1%). Most patients (75%) received ≥4 docetaxel cycles, half received ≥6 cycles, 25% received ≥8 cycles and 10% received ≥10 cycles. Increased tumor mass, with/without new bone lesions or rising PSA, was the most common reason for docetaxel discontinuation (74% of patients).

**Conclusions:** Concordant with guidelines, docetaxel and prednisone was the preferred first-line chemotherapy regimen in CRPC patients reviewed for this study. However, one quarter of patients did not initiate docetaxel until ≥6 months after CRPC diagnosis and total exposure varied considerably, with only 10% receiving ≥10 cycles. Future studies are needed to describe specific reasons explaining timing of docetaxel initiation and duration of exposure in some CRPC patients.

This is novel research into important methodological concerns often overlooked up until now.

## BACKGROUND

Prostate cancer is the most commonly diagnosed malignancy among men in the United States and is the second-leading cause of cancer-related death in males.[1,2] Approximately 180,000 newly diagnosed cases of prostate cancer occur each year in the United States, and the number of prevalent cases exceeds 2 million.[3] Although only 4% of incident cases are diagnosed after the cancer has already metastasized, up to 50% of patients diagnosed with localized disease will eventually progress to having metastatic prostate cancer.[3]

Growth and proliferation of prostate cancer cells is dependent on the androgen receptor (AR), which is expressed in nearly all prostate cancers.[4] Depriving prostate cancer cells of testosterone reverses AR-dependent growth and proliferation. Androgen deprivation therapy (ADT), which pharmacologically reduces testosterone to castrate levels, is therefore the usual first-line treatment for most patients with prostate cancer. Although the majority of men with prostate cancer will initially respond to ADT, ARs eventually reactivate and progression to castration-resistant prostate cancer (CRPC) almost always occurs.[5-7] Compared with castration-sensitive prostate cancer, the prognosis for CRPC is poor, survival is reduced, and until 2004 available treatments were limited mainly to watchful waiting or symptomatic relief of bone metastases.[8,9]

In 2004, docetaxel in combination with prednisone was approved in the United States for the treatment of patients with CRPC, as it demonstrated a statistically significant, although modest, improvement in survival over the previous standard regimen of mitoxantrone plus prednisone.[10] Since then, clinical guidelines have shifted to recommend docetaxel plus prednisone as the preferred first-line treatment for CRPC.[11] While newer treatments have since been approved for CRPC in the United States, these new alternatives are recommended either for asymptomatic CRPC (sipuleucel-T) or as second-line treatment (abiraterone acetate, enzalutamide, cabazitaxel).[12] Although docetaxel-based chemotherapy in patients with CRPC improves median survival by approximately 3 months, patients eventually experience further disease progression, typically within 7 months after treatment initiation.[13,14]

Following the emergence of docetaxel-based chemotherapy as the new standard of care for CRPC, surprisingly little has been reported on its patterns of use in routine practice. Because treatment approaches are often influenced by factors that are intentionally controlled in clinical trials, analyses of observational data from a real-world population may provide an understanding of how treatment is being implemented in actual clinical practice. Such information may aid clinicians, payers and other stakeholders in the provision of optimal CRPC care. To help understand these treatment patterns, we conducted a retrospective study of medical records for patients from across the United States who received a docetaxel-based regimen for the treatment of CRPC.

## MATERIALS AND METHODS

Medical records from 394 patients with CRPC who were treated in the United States were reviewed. Data were collected from physicians using a standardized case report form (CRF). According to a predefined physician enrollment quota, a total of 48 physicians from oncology practices and 8 from urology practices were recruited for the chart abstraction. The selected physicians were required to have an annual prostate cancer caseload of at least 10 patients and to have between 2 and 35 years of experience in clinical practice.

Patients who met the following criteria were selected for the study:

- CRPC first diagnosed between 2004 and 2010, with castration resistance defined by disease progression as evidenced by steadily rising prostate-specific antigen (PSA) values (i.e., three consecutive increases or

- at least a 50% increase over the nadir PSA) or an increase in tumor mass (per X-ray, CT scan, or MRI) despite castrate levels of testosterone (<50 ng/dL)[15,16]
- Age at least 18 years at CRPC diagnosis
- Received a docetaxel-based regimen (minimum of 2 cycles) for treatment of CRPC
- Discontinued docetaxel due to rising PSA, progression of bone lesions, or progression of nodal or visceral metastases. (Patients who discontinued docetaxel due to adverse events were not eligible.)
- Continuous chart history (i.e., follow-up) until at least 3 months after docetaxel discontinuation

Patients were selected using a "convenience" sampling design, whereby charts were screened and reviewed by the participating physicians in consecutive retrospective order of CRPC diagnosis date until a minimum case quota (at least 3, but not more than 10 patients) was met for each physician. The intent of this data collection approach was to facilitate gathering of the most recent data available. From the collected chart data, we evaluated patient demographics, prevalent comorbidities[17], characteristics of the docetaxel regimen initiated, and other treatments received until docetaxel discontinuation; a review of additional cancer-directed treatments received after docetaxel discontinuation was also performed.

The research presented in this report was conducted with Institutional Review Board (IRB) approval in accordance with the Helskinki Declaration on the protection of human subjects. The research organization conducting this study, RTI Health Solutions, a business unit of RTI International, holds a Federal-Wide Assurance (FWA #3331) from the US Department of Health and Human Services (DHHS) Office for Human Research Protections (OHRP) that allows for the review and approval of human subjects protocols through internal IRB committees. These ethics committees review research studies to ensure adherence to appropriate regulations that govern human subjects research, including 45 CFR 46, 21 CFR 50 and 56, and with all applicable International Conference on Harmonization provisions, including the Helsinki Declaration.

## RESULTS

From the 48 oncologists participating in the study, data were collected on a total of 344 patients meeting the noted inclusion criteria. From the 8 urologists in the study, data were collected on 50 patients, resulting in a total sample of 394 patients. In the overall sample, mean [±SD] age was 66.5 [8.9] years and the majority (62.7%) were white (Table 1). Most patients included in the review initiated docetaxel between 2008 and 2010. Medicare was the most common insurance type (57.9% of all patients), followed by commercial insurance (26.9% of all patients). Despite having a similar age distribution as patients treated by oncologists, a substantially higher proportion of those treated by urologists were covered by Medicare (82.0% of patients). Existing comorbidity burden independent of prostate cancer was similar between patients treated by oncologists and urologists, with diabetes, pulmonary disorders, and congestive heart failure being among the most common comorbid conditions.

**Table 1.** Patient Characteristics, by Specialty of Treating Physician

| | All Patients (N = 394) | | Physician Specialty | | | |
|---|---|---|---|---|---|---|
| | | | Oncology (N = 344) | | Urology (N = 50) | |
| | N | % | N | % | N | % |
| **Age at CRPC Diagnosis** | | | | | | |
| Mean (Std Dev) | 66.52 (8.92) | | 66.35 (9.14) | | 67.72 (7.23) | |
| Median (Min, Max) | 67 (18, 93) | | 67 (18, 93) | | 66.5 (55, 85) | |
| **Race/Ethnicity** | | | | | | |
| White | 247 | 62.69 | 208 | 60.47 | 39 | 78.00 |
| Black | 109 | 27.66 | 98 | 28.49 | 11 | 22.00 |
| Hispanic | 25 | 6.35 | 25 | 7.27 | --- | --- |
| Other | 13 | 3.30 | 13 | 3.78 | --- | --- |
| **Geographic Location** | | | | | | |
| Northeast | 89 | 22.59 | 89 | 25.87 | --- | --- |
| South | 135 | 34.26 | 96 | 27.91 | 39 | 78.00 |
| Midwest | 107 | 27.16 | 96 | 27.91 | 11 | 22.00 |
| West | 43 | 10.91 | 43 | 12.50 | --- | --- |
| Unknown | 20 | 5.08 | 20 | 5.81 | --- | --- |
| **Primary Insurance Type** | | | | | | |
| Commercial | 106 | 26.90 | 99 | 28.78 | 7 | 14.00 |
| Medicare | 228 | 57.87 | 187 | 54.36 | 41 | 82.00 |
| Medicaid | 34 | 8.63 | 33 | 9.59 | 1 | 2.00 |
| Other | 4 | 1.02 | 4 | 1.16 | --- | --- |
| Unknown | 22 | 5.58 | 21 | 6.10 | 1 | 2.00 |
| **Year of CRPC Diagnosis** | | | | | | |
| 2004 | 17 | 4.31 | 16 | 4.65 | 1 | 2.00 |
| 2005 | 40 | 10.15 | 35 | 10.17 | 5 | 10.00 |
| 2006 | 27 | 6.85 | 19 | 5.52 | 8 | 16.00 |
| 2007 | 38 | 9.64 | 25 | 7.27 | 13 | 26.00 |
| 2008 | 67 | 17.01 | 56 | 16.28 | 11 | 22.00 |
| 2009 | 122 | 30.96 | 116 | 33.72 | 6 | 12.00 |
| 2010 | 83 | 21.07 | 77 | 22.38 | 6 | 12.00 |
| **Charlson Comorbidity Score** | | | | | | |
| Mean (Std Dev) | 1.57 (1.65) | | 1.55 (1.64) | | 1.72 (1.73) | |
| Median (Min, Max) | 1 (0, 8) | | 1 (0, 8) | | 1 (0, 6) | |

CRPC: castration-resistant prostate cancer

**Table 1.** Patient Characteristics, by Specialty of Treating Physician - continued

| | All Patients (N = 394) | | Physician Specialty | | | |
|---|---|---|---|---|---|---|
| | | | Oncology (N = 344) | | Urology (N = 50) | |
| | N | % | N | % | N | % |
| **Charlson Comorbidities** | | | | | | |
| Cerebrovascular Disease | 36 | 9.14 | 25 | 7.27 | 11 | 22.00 |
| Chronic Pulmonary Disease* | 93 | 23.60 | 86 | 25.00 | 7 | 14.00 |
| Congestive Heart Failure | 61 | 15.48 | 48 | 13.95 | 13 | 26.00 |
| Connective Tissue Disease | 13 | 3.30 | 12 | 3.49 | 1 | 2.00 |
| Dementia | 12 | 3.05 | 11 | 3.20 | 1 | 2.00 |
| Hemiplegia | 2 | 0.51 | 2 | 0.58 | --- | --- |
| HIV/AIDS | 1 | 0.25 | 1 | 0.29 | --- | --- |
| Leukemia | 3 | 0.76 | 3 | 0.87 | --- | --- |
| Malignant Lymphoma | 3 | 0.76 | 3 | 0.87 | --- | --- |
| History of Myocardial Infarction | 53 | 13.45 | 46 | 13.37 | 7 | 14.00 |
| Peripheral Vascular Disease | 51 | 12.94 | 42 | 12.21 | 9 | 18.00 |
| Ulcer Disease | 16 | 4.06 | 16 | 4.65 | --- | --- |
| Diabetes | 108 | 27.41 | 93 | 27.03 | 15 | 30.00 |
| Liver Disease | 10 | 2.54 | 10 | 2.91 | --- | --- |
| Renal Disease | 61 | 15.48 | 52 | 15.12 | 9 | 18.00 |
| Solid Tumor (Other than Prostate) | 3 | 0.76 | 2 | 0.58 | 1 | 2.00 |
| Ulcer Disease | 16 | 4.06 | 16 | 4.65 | --- | --- |
| Diabetes | 108 | 27.41 | 93 | 27.03 | 15 | 30.00 |
| Liver Disease | 10 | 2.54 | 10 | 2.91 | --- | --- |
| Renal Disease | 61 | 15.48 | 52 | 15.12 | 9 | 18.00 |
| Solid Tumor (Other than Prostate) | 3 | 0.76 | 2 | 0.58 | 1 | 2.00 |

*Includes bronchitis, chronic bronchitis, emphysema, asthma, bronchiectasis, extrinsic allergic alveolitis, chronic airway obstruction (not elsewhere classified), pneumoconioses and other lung diseases due to external agents, and chronic respiratory conditions due to fumes and vapors.

Among all patients, most (78.9%) initiated docetaxel with prednisone, while 18.5% initiated docetaxel alone and 2.6% initiated docetaxel with other drugs (Figure 1). However, a substantially higher proportion of patients treated by urologists (32.0%) initiated docetaxel as monotherapy; most of the remaining sample from urologists (66%) initiated docetaxel with prednisone. Among all patients combined, other non-chemotherapy treatments used in combination with docetaxel were hormonal therapy (22.8%), radiotherapy (17.3%), surgery (4.1%), and non-prednisone immunotherapy (0.5%) (Figure 2). In the 50 patients treated by urologists, a substantially higher proportion (58.0%) as compared with those treated by oncologists (17.7%) received additional hormone therapy (most commonly bicalutamide or leuprolide) in combination with docetaxel.

**Figure 1.** Docetaxel-Based Regimen Initiated, by Specialty of Treating Physician

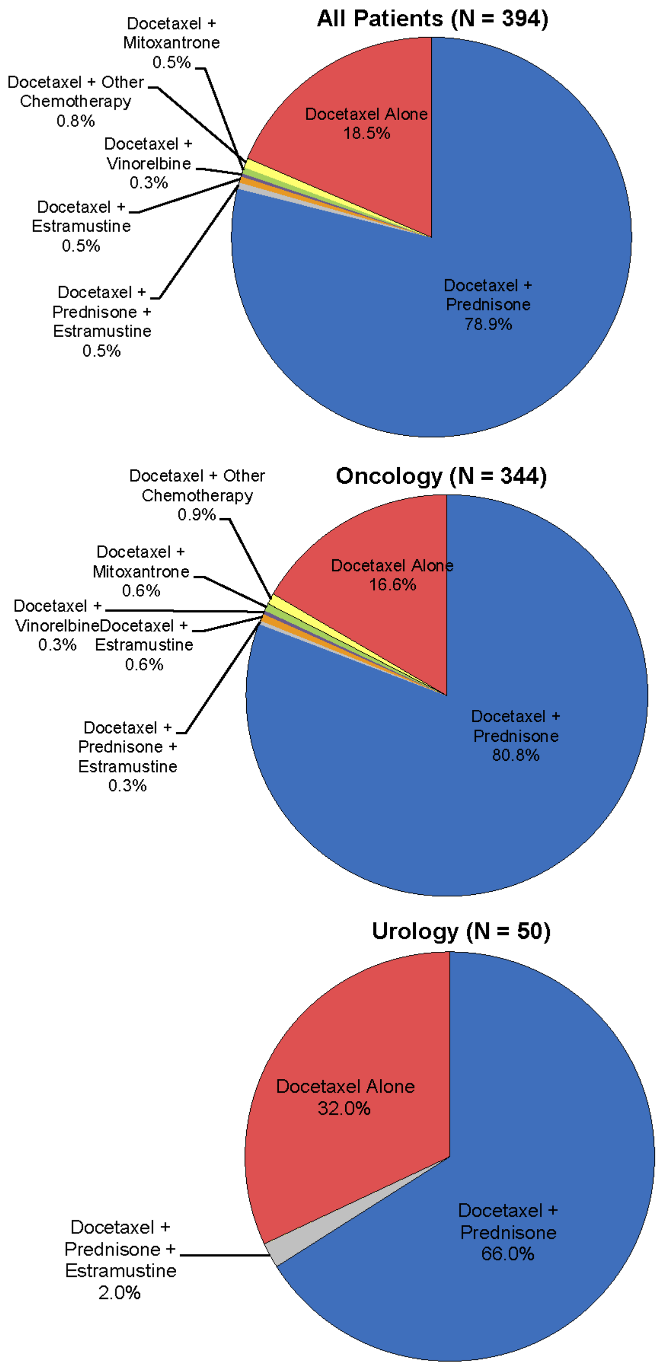

**Figure 2.** Other Cancer-Directed Treatments Received in Combination with Docetaxel, by Specialty of Treating Physician

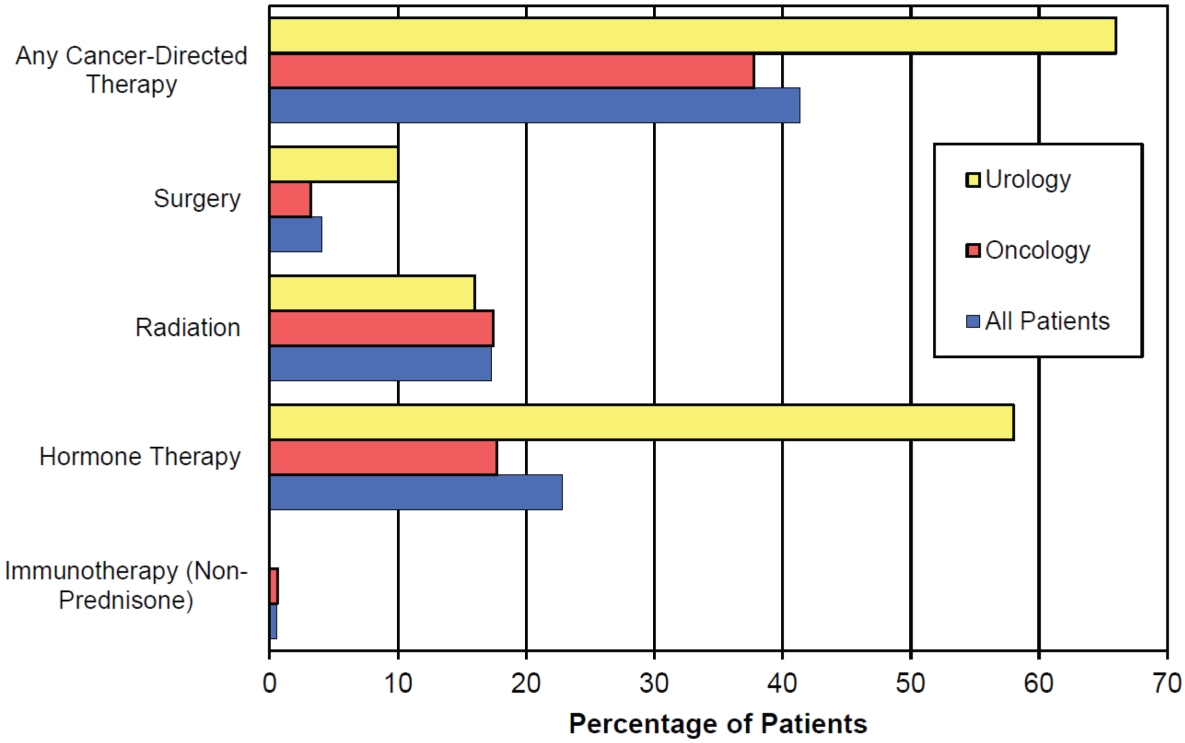

When docetaxel was used, it was initiated as first-line CRPC treatment in nearly all patients (90.7% of patients treated by oncologists, 100% of patients treated by urologists) at a median starting dose of 75 mg/m2 (Table 2). Half (50%) of all patients initiated docetaxel within 1 month after CRPC diagnosis, regardless of the treating physician's specialty, while 25% started at least 6 months later. Among patients treated by urologists, however, 25% of patients waited at least 11 months after CRPC diagnosis to start docetaxel. Examining all patients combined, most (75%) received at least four docetaxel cycles, half (50%) received at least 6 cycles (median), 25% received at least 8 cycles and 10% received at least 10 cycles. Patients treated by urologists received substantially fewer docetaxel cycles (median = 3) compared with those treated by oncologists (median = 6).

Among all patients combined, mean [±SD] and median time to docetaxel discontinuation was 7.5 [5.6] and 6 months, respectively (Table 3). Docetaxel duration was somewhat higher for patients treated by urologists (mean [±SD] and median time to discontinuation of 9.1 [6.3] and 7 months, respectively) as compared with patients treated by oncologists (mean [±SD] and median time to discontinuation of 7.3 [5.3] and 6 months, respectively). Among all patients, increased tumor mass (with or without new bone lesions or rising PSA) was the most common reason for docetaxel discontinuation (74% of patients). Approximately 10% of patients discontinued following 10 cycles of docetaxel as described in the TAX327 protocol.[13]

After docetaxel discontinuation, slightly more than half (50.5%) of the study sample received some form of additional cancer-directed therapy during the follow-up time available for study, most commonly additional chemotherapy (Figure 3). In order of magnitude among all patients, the post-docetaxel prevalence of additional cancer-directed therapies was chemotherapy (33.0%), radiation therapy (14.2%), hormone therapy (9.1%), immunotherapy (2.0%), surgery (1.3%), and angiogenesis inhibitors (0.3%). By physician specialty, a higher proportion of patients treated by oncologists received further cancer-directed therapy as compared with patients treated by urologists (54% vs. 28%).

**Table 2. Docetaxel Initiation Characteristics, by Specialty of Treating Physician**

| | All Patients (N = 394) | | Physician Specialty | | | |
|---|---|---|---|---|---|---|
| | | | Oncology (N = 344) | | Urology (N = 50) | |
| | N | % | N | % | N | % |
| **Initiated Docetaxel as First-Line Therapy for CRPC?** | | | | | | |
| Yes | 362 | 91.88 | 312 | 90.70 | 50 | 100.00 |
| No | 32 | 8.12 | 32 | 9.30 | --- | --- |
| **Time (Months) to Initiation After CRPC Diagnosis** | | | | | | |
| Mean (Std Dev) | 6.73 (12.92) | | 6.88 (13.41) | | 5.72 (8.92) | |
| Min, Max | 0, 70 | | 0, 70 | | 0, 32 | |
| 1st Percentile | 0 | | 0 | | 0 | |
| 5th Percentile | 0 | | 0 | | 0 | |
| 10th Percentile | 0 | | 0 | | 0 | |
| 25th Percentile | 0 | | 0 | | 0 | |
| 50th Percentile (Median) | 1 | | 1 | | 1 | |
| 75th Percentile | 6 | | 6 | | 11 | |
| 90th Percentile | 22 | | 24 | | 19 | |
| 95th Percentile | 38 | | 39 | | 30 | |
| 99th Percentile | 60 | | 60 | | 32 | |
| **Dose at Initiation (mg/m2)** | | | | | | |
| Mean (Std Dev) | 69.99 (18.76) | | 70.22 (16.47) | | 68.48 (30.35) | |
| Median (Min, Max) | 75 (4, 162) | | 75 (20, 162) | | 75 (4, 100) | |
| **Number of Cycles Received** | | | | | | |
| Mean (Std Dev) | 6.75 (4.84) | | 7.24 (4.97) | | 3.42 (1.33) | |
| Min, Max | 1, 50 | | 1, 50 | | 2, 8 | |
| 1st Percentile | 2 | | 2 | | 2 | |
| 5th Percentile | 2 | | 3 | | 2 | |
| 10th Percentile | 3 | | 3 | | 2 | |
| 25th Percentile | 4 | | 4 | | 3 | |
| 50th Percentile (Median) | 6 | | 6 | | 3 | |
| 75th Percentile | 8 | | 8 | | 4 | |
| 90th Percentile | 10 | | 12 | | 6 | |
| 95th Percentile | 14 | | 15 | | 6 | |
| 99th Percentile | 25 | | 25 | | 8 | |

CRPC: castration-resistant prostate cancer

**Table 3.** Docetaxel Discontinuation Characteristics, by Specialty of Treating Physician

| | All Patients (N = 394) | | Physician Specialty | | | |
|---|---|---|---|---|---|---|
| | | | Oncology (N = 344) | | Urology (N = 50) | |
| | N | % | N | % | N | % |
| **Time (Months) to Discontinuation After Initiation** | | | | | | |
| Mean (Std Dev) | 7.51 (5.49) | | 7.28 (5.34) | | 9.11 (6.26) | |
| Median (Min, Max) | 6 (2, 40) | | 6 (2, 40) | | 7 (3, 35) | |
| **Reason for Discontinuation** | | | | | | |
| Rising PSA Only | 9 | 2.28 | 9 | 2.62 | --- | --- |
| Rising PSA + New Bone Lesions | 9 | 2.28 | 8 | 2.33 | 1 | 2.00 |
| Rising PSA + Increased Tumor Mass | 34 | 8.63 | 27 | 7.85 | 7 | 14.00 |
| Rising PSA + New Bone Lesions + Increased Tumor Mass | 1 | 0.25 | --- | --- | 1 | 2.00 |
| New Bone Lesions Only | 23 | 5.84 | 22 | 6.40 | 1 | 2.00 |
| New Bone Lesions + Increased Tumor Mass | 140 | 35.53 | 106 | 30.81 | 34 | 68.00 |
| Increased Tumor Mass Only | 118 | 29.95 | 116 | 33.72 | 2 | 4.00 |
| Completed planned regimen (≥10 cycles)* | 39 | 9.90 | 39 | 11.34 | --- | --- |
| Reason for Discontinuation Unknown | 21 | 5.33 | 17 | 4.94 | 4 | 8.00 |

*Per TAX327 protocol, a complete regimen consists of 10 cycles PSA: prostate-specific antigen

**Figure 3.** Additional Cancer-Directed Treatments Received After Docetaxel Discontinuation, by Specialty of Treating Physician

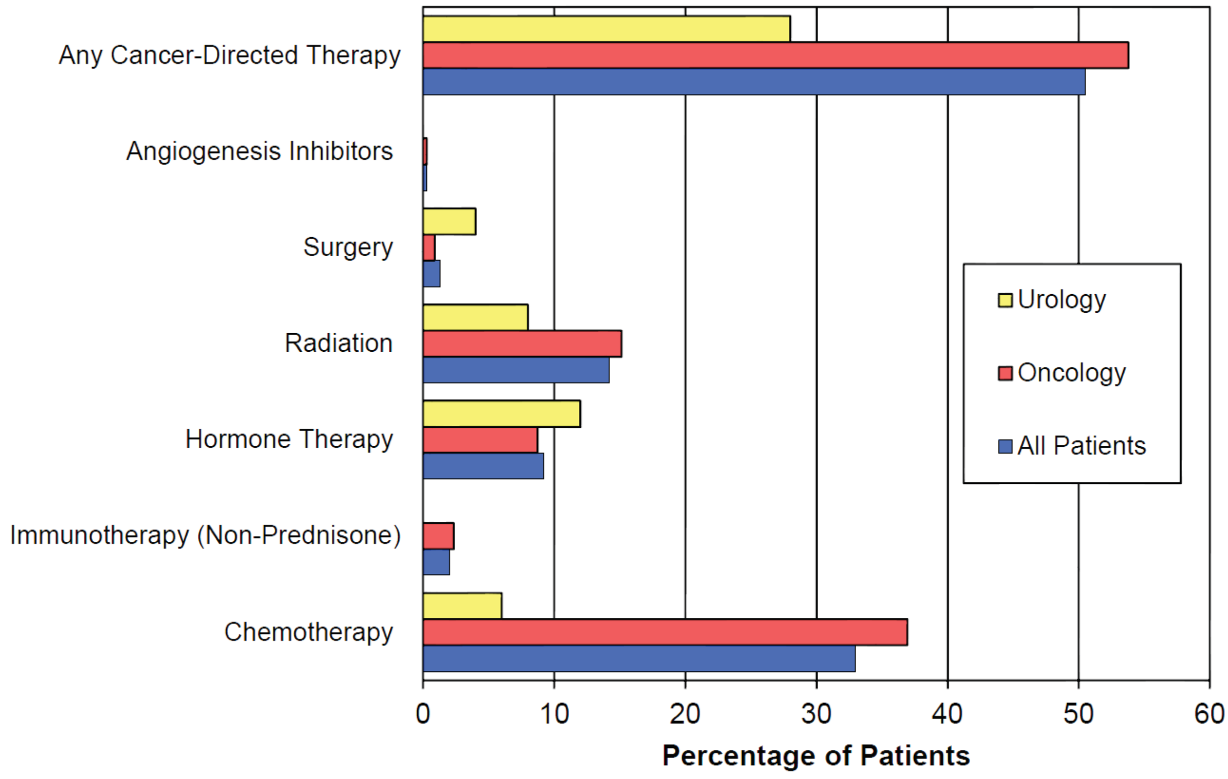

## DISCUSSION

We conducted a retrospective analysis of medical records for 394 patients from across the United States who received a docetaxel-based regimen for the treatment of CRPC. Concordant with current guidelines, docetaxel plus prednisone when it is used, it is most often in the first line setting. However, one quarter of patients did not initiate docetaxel until at least 6 months after CRPC diagnosis and total exposure varied considerably, with only 10% of patients able to complete 10 cycles as intended in the TAX327 protocol, and only 50% completing 6 cycles. Approximately half of all patients received no further cancer-directed therapy (chemotherapy or other treatment) after discontinuation of docetaxel, which highlights the well-documented unmet need in this population.

As noted earlier, few observational studies have documented the real-world treatment patterns associated with CRPC. To our knowledge, only one previous study[14] specifically assessed docetaxel-related treatment patterns among patients receiving docetaxel as first-line therapy for CRPC. In this study, a retrospective chart review was conducted on 88 CRPC patients at a single institution who were treated with first-line docetaxel from August 2005 to June 2007, with follow-up until February 2008. Patients included in the review were somewhat older (median age = 71 years) compared with our study sample. Similar to our study, Chin et al.[14] observed a total docetaxel exposure (median = 7 cycles, vs. 6 cycles in our study) that was considerably less than the 10 cycles planned in the TAX327 protocol. Following docetaxel discontinuation, Chin et al.[14] found that 41% of patients went on to receive additional chemotherapy, whereas 33% of our study sample did so. Among those who initiated second-line chemotherapy, Chin et al.[14] found mitoxantrone to be the predominant choice (89% of patients); in our study, which captured data after the 2010 launch of cabazitaxel, only 37% of patients receiving second-line chemotherapy were treated with mitoxantrone, while 38% were treated with cabazitaxel (tabular data available upon request). Similar to Chin et al.[14], who reported a median second-line chemotherapy exposure of 3.5 cycles, we found a median exposure of 4 cycles.

Our study also provides new insights regarding differences in CRPC treatment patterns among urologists as compared with oncologists. We found that duration of docetaxel treatment among patients managed by urologists was longer as compared with patients managed by oncologists, but urologists administered fewer docetaxel cycles as compared with oncologists. Although reasons for this difference were not determined, this suggests that, compared with oncologists, the urologists participating in our study may have more frequently delayed cycles, that delays were longer on average, or that therapy was discontinued earlier. Further research may be warranted to investigate whether patients treated by urologists, or the urologists themselves, have specific characteristics that influence the duration of therapy and number of chemotherapy cycles administered.

This study was subject to several limitations. As an inherent limitation of retrospective chart abstractions, subjects selected for study inclusion represented a "convenient" sample. Our findings therefore may not be generalizable to the overall CRPC population in the United States. The study findings also are not generalizable to the overall prostate cancer population, many of whom never need systemic therapy. Second, while no time limit was imposed on physicians for completion of the chart review, to ensure that they responded as fully and accurately as possible, the CRF was designed to limit physicians' time burden and therefore could not capture all relevant study variables that may have been of interest. To this extent, our review was limited only to treatment patterns related to docetaxel and did not address overall practice patterns for CRPC or associated outcomes such as survival. One recent review addresses this gap, noting that only 37% of all patients with CRPC receive chemotherapy, with the remaining 63% receiving only steroids and supportive care.[8] In addition, excluding patients who discontinued docetaxel due to non-cancer-related adverse events and requiring at least 3 months of follow-up after docetaxel discontinuation may have resulted in overestimation of the time on docetaxel treatment for all patients with CRPC who initiate this therapy. Finally, owing to possible truncation of follow-up time for patients who entered the study during later years (i.e., closer to the time of data collection), we may have underestimated the proportion who eventually received additional therapies.

## CONCLUSIONS

Despite the noted limitations, our analysis provides new data on the use of docetaxel in routine practice for the treatment of CRPC. In addition to finding that delayed initiation after CRPC diagnosis was common, we found that most patients did not complete 10 cycles of chemotherapy, and a low rate of further treatment after discontinuation of docetaxel. Our study therefore confirms and further highlights the well-documented unmet need in patients with CRPC. Future studies are needed to determine the specific reasons for delays in initiating docetaxel, sub-maximal docetaxel exposure, and the limited use of second- and third-line therapies in the post-docetaxel setting among CRPC patients.

## CONFLICT OF INTEREST DECLARATION

The authors wish to disclose that this study and the writing and preparation of this manuscript were funded in full by Bristol-Myers Squibb (BMS), which is conducting clinical research in the area of prostate cancer. RTI Health Solutions was the consulting firm hired and paid by BMS to collaborate in developing the study design, conducting the research study and analyses, and drafting the first version this manuscript. KLD and JAK are employees RTI Health Solutions. BG was an employee of BMS at the time this study was conducted. TZ is a current employee of BMS. The publication of this study's results is not contingent upon the sponsor's approval or subject to censorship of the manuscript.

### Acknowledgements

The authors wish to acknowledge the critical contributions of Constanze Cholmakow-Bodechtel and Tamara Bendelack to this study. As employees of Kantar Health, Ms. Cholmakow-Bodechtel and Ms. Bendelack led and executed all fieldwork activities for the collection of the medical record data required for this study.
